# The ‘sugar tax’ in Bermuda: a mixed methods study of general population and key stakeholder perceptions

**DOI:** 10.1186/s12889-022-13945-9

**Published:** 2022-08-16

**Authors:** Kelsey K. Case, Elisa Pineda, Jack Olney, Alexa Blair Segal, Franco Sassi

**Affiliations:** 1 School of Public Health, Imperial College London, Norfolk Place, London, W2 1PG UK; 2 Centre for Health Economics & Policy Innovation, Imperial College Business School, Imperial College London, London, UK

**Keywords:** Sugar tax, Discretionary foods tax, Health tax, Mixed-methods analysis, Bermuda

## Abstract

**Background:**

Taxes on discretionary foods and sugar-sweetened beverages have emerged as a strategy for health promotion. Between 2018–2019, the Bermuda government introduced a phased tax on imported sugar-sweetened beverages, confectionery, products containing cocoa and pure sugar, and eliminated import duties on select healthy food items. The aim of this study was to conduct an mixed methods evaluation of perceptions of the tax among the general population and key stakeholders.

**Methods:**

We conducted a survey of the general population (*N* = 400), and semi-structured interviews with key informants (*N* = 14) from the government, food and beverage, and health sectors to understand awareness, acceptability, and perceived impact of the tax after implementation. Survey data was analysed using thematic analysis, summary statistics, and Chi-squared tests. Key informant interviews were analysed using the framework method.

**Results:**

General population respondents had high awareness of the sugar tax (94%) but low awareness of the healthy food subsidy (32%). Most respondents (67%) felt the tax was not an appropriate way to motivate healthier consumption due to beliefs the tax would not be effective (44%), and because of the high price of healthy food (20%). However, nearly half (48%) reported consuming fewer taxed products, primarily for health reasons but also motivated by price increases. Key informants indicated there was high awareness but limited understanding of the tax policy. Informants expressed support for taxation as a health promotion strategy, conditional on policy implementation. The lack of clear price differentiation between taxed and un-taxed products and the absence of accompanying health education were key factors believed to affect the impact of the tax. No informants were aware of use of tax revenues for health purposes and tax revenue was reportedly re-directed to other priorities after implementation.

**Conclusions:**

There was high awareness, but limited acceptability of the Bermuda sugar tax as implemented. Clarity in the tax policy, appropriateness of the tax mechanism, and use of revenue in alignment with the tax aim are critical components for acceptance. The absence of complementary education and health promotion affected acceptance and may limit potential health impacts. The lessons learned in Bermuda can inform similar policies in other settings.

**Supplementary Information:**

The online version contains supplementary material available at 10.1186/s12889-022-13945-9.

## Background

Overweight and obesity are risk factors for chronic non-communicable diseases such as diabetes, cardiovascular disease and cancer, and a cause of premature mortality. Globally, the prevalence of obesity nearly tripled between 1975 and 2016 [1], making it an important public health priority. Taxes and other fiscal incentives on food and non-alcoholic beverages have been proposed as a strategy to influence purchasing behaviours and reduce morbidity and mortality from obesity, diabetes, and other non-communicable diseases [2]. A growing body of evidence shows the effectiveness of fiscal policies for health. The taxation of alcohol and tobacco is now a well-established approach to health promotion [3], while fiscal policies for diet are being increasingly used [4]. A growing number of local and national jurisdictions have now introduced taxes on sugar-sweetened beverages and discretionary foods [5].

Bermuda is a British Overseas Territory situated in the Atlantic Ocean with a land area of 21 square miles and a population just under 64,000 people [6]. The majority of food in Bermuda is imported from overseas [7]. Compared to other OECD (Organisation for Economic Co-operation and Development) countries, Bermuda has disproportionately high rates of diabetes, overweight and obesity, with the latter two affecting an estimated 75% of the population [8, 9]. This has serious implications for public health and current and future health expenditure.

A sugar tax was proposed in 2017 as a new approach to address the country’s unhealthy weight with the aims to raise awareness about healthy eating and deter the purchase of sugary items. Funds raised from the tax would financially support and enhance health promotion and community education [10]. An online public consultation held between 4 January 2018 and 1 March 2018 found general support for a sugar tax, support for a high level of tax, and beliefs that the tax could lead to behaviour change. The key concerns identified were regarding tax implementation and education [10].

On 1 October 2018, the Bermuda government introduced a 50% import duty on imported confectionery, sugar-sweetened beverages, pure sugar and dilutables (e.g. syrups). This resulted in a 14.5–50% increase compared to pre-tax import duties. On 1 April 2019, the level of the tax was increased to 75%, and the tax base was expanded to include food products containing cocoa. Drinks without added sugar and 100% fruit juices were excluded from the tax. Import duties on select healthy food items were eliminated. Between 1 October 2018 and 31 December 2019, over US$ 5.4 million was raised in revenue from the sugar tax [11].

The use of taxation as public health policy is a sensitive topic with complex political, social, and economic dynamics. Public and key stakeholder perceptions, and the lessons learned from countries that have implemented these taxes, are critical to understand to inform the design of effective policies. The Bermuda sugar tax is notable in that it goes beyond taxing just sugar-sweetened beverages, at a tax level that is among the highest in the world, and includes a healthy food subsidy. The purpose of this study was to understand how the sugar tax in Bermuda was perceived after policy implementation. The specific objectives were to examine beliefs and perceptions surrounding awareness, acceptability and impact the tax among 1) the general population, and 2) key stakeholders involved in, or affected by, the tax. A better understanding of these perspectives and experiences can further inform the tax policy implemented in Bermuda, and the design and implementation of health taxes in other countries. This study is likely particularly relevant for other small island nations, but the lessons learned may be more broadly applicable in other settings.

## Methods

A mixed methods study was conducted consisting of a general population survey and key informant interviews. The methods are described below.

### General population survey

General population perceptions of the sugar tax in Bermuda were assessed through the Bermuda Omnibus Survey. This is a quarterly survey of general population perspectives on political, economic and social issues [12]. We designed and commissioned a set of six open- and closed-ended questions for the Bermuda Omnibus Survey to assess perceptions regarding awareness, acceptability, and impact of the sugar tax (Additional File 1). The questions were revised, validated and piloted by the Total Research Group, the local market research company administering the survey. The finalised questions were incorporated into the Bermuda Omnibus Survey, First Quarter 2020.

The survey was undertaken using computer-assisted telephone interviews. Data were collected from a nationally representative sample of 400 residents aged 18 years and older. Participants were recruited via random sampling from landlines combined with random digit dialling sampling for mobile phones. The sample was stratified to represent the Bermuda population across key characteristics including age, sex, race, and parish of residence. The survey was conducted between 4–18 March 2020, prior to COVID-19 pandemic restrictions implemented in Bermuda. Unaided, open-ended responses were coded into thematic categories for analysis. Results were weighted for population representativeness at the national level. To understand if there were variations by sociodemographic variables, the association of the awareness of the sugar tax, awareness of the fruit and vegetable subsidy, and acceptability of the sugar tax by age, sex, race, and household income were independently tested using Pearson’s chi-square tests. Analyses were conducted in Stata 17.0 with a *p*-value < 0.05 as the cutoff for statistical significance. Post-hoc analyses were conducted on significant chi-square results using a z-test on the adjusted residuals with Bonferroni correction.

### Key informant interviews

Perceptions of the sugar tax among key stakeholders were assessed using semi-structured interviews. The topics addressed included awareness, acceptability, and perceived impact of the tax. Open-ended questions were used to allow the findings to be driven by the participant and their own experiences (Additional File 1). Purposive sampling was used to obtain a sample of key individuals engaged in the process of proposing, developing, or implementing the sugar tax, businesses impacted by the tax, or individuals from health or health promotion fields. Snowball sampling was used to identify additional participants to complete the sampling frame. The sampling frame was designed to provide a wide range of perspectives. Key informants included individuals from ‘government’ (civil servants, public officers, members of Parliament), ‘health’ (health practitioners, nutritionists, health promotion advocates), and ‘food and beverage’ (restaurants, grocery stores, wholesalers, convenience stores, beverage distributors). Sampling saturation occurred when new information was no longer identified.

Participants were recruited via email, provided with the study information sheet, and invited to participate. Interviews were conducted in English via an online teleconference platform (Zoom) to reduce potential risk of SARS-CoV-2. All participants were over 18 years of age. Informed consent was obtained from all participants. No renumeration was provided for participation. Interviews were conducted between 3 August 2020 and 11 September 2020, just over 16 months after full implementation of the sugar tax. Interviews lasted approximately 45 min and were recorded with permission and transcribed. Transcriptions were reviewed against the recording for accuracy then anonymised and uploaded into NVivo 12 Plus for coding and analysis. All data were securely stored. Data were collected, coded, and analysed by a single researcher (KKC). A second researcher (EP) coded a sub-sample of the interviews (2/14) to assess inter-rater reliability (94.3% agreement, Kappa coefficient 0.50).

The qualitative data were analysed using the framework method and incorporated data analysis principles from grounded theory. The framework method is an approach developed by researchers in the United Kingdom to analyse qualitative data applied to policy research [13], and now frequently used in policy and health research [14]. It is based on a common set of principles which comprise qualitative analysis but notably includes the development of a matrix to review, analyse and summarize the data [14]. Grounded theory is a qualitative research method that is grounded in the data systematically collected. Codes and themes emerge from the data and opposed to being pre-defined [15]. This study utilised the framework method but incorporated inductive coding, memoing, and constant comparative analysis, which are central grounded theory, in the data analysis procedure.

Interview transcripts were first coded line-by-line using an inductive approach with initial codes generated from the data through open coding. Initial codes were grouped into broader categories (axial coding) and refined into emerging themes. As a more defined theme and sub-theme structure emerged, the categorisation was compared with published studies which resulted in further refinement in the codes and additional deductive coding conducted across all transcripts. Data collection and analysis occurred concomitantly and as an iterative process. Emergent findings were cross-referenced in subsequent interviews and triangulated with government and policy documents, sugar tax consultation documents, and local news media. When the coding and analytical framework was complete, the results were summarised in a framework matrix. Cross and between group comparisons of the results were conducted for each theme and sub-theme. Findings were discussed and reviewed by the entire research team.

### Reflexivity statement

The researcher conducting all interviews (KKC) was both a Bermudian resident and part of a scientific research team from Imperial College London. The researcher has substantial experience conducting qualitative research in public health, continually sought to maintain objectivity, and was mindful of their own perspectives and perceptions and the potential implications on the research. The study sampling frame was specifically designed to capture diversity in perspectives and all findings were reviewed and discussed with the entire research team.

### Ethics statement

Both Imperial College London and the Bermuda Hospitals Board granted ethical approval for this study. Informed consent was obtained from all participants for participation in the study, recording the interview and publication of the findings. The Standards for Reporting Qualitative Research guidelines [16] were adhered to for this study and are documented in Additional File 1, Table S1.

## Results

### General population survey results

Four hundred adult Bermudian residents were recruited for the Bermuda Omnibus Survey. The sample recruited was broadly representative of the general population by sex, household income, age, race, Bermudian status, and parish of residence, Table 1. Results were weighted for national representativeness.

**Table 1:** Sociodemographic characteristics of survey participants, Bermuda Omnibus Survey, First Quarter 2020, *N* = 400

	**Unweighted sample size N (%)**	**Weighted sample size N (%)**
**Sex**		
Male	176 (44)	190 (48)
Female	224 (56)	210 (52)
**Household income**		
< $75 K	130 (32)	140 (35)
$75,000 – < $150,000	133 (33)	134 (34)
≥ $150,000	106 (27)	97 (24)
Don’t know/no answer	31 (8)	29 (7)
**Age**		
18–34	65 (16)	91 (23)
35–54	167 (42)	176 (44)
55 +	168 (42)	133 (33)
**Race**		
Black	177 (44)	198 (50)
White	153 (38)	118 (30)
Other	39 (10)	53 (13)
No answer	31 (8)	31 (8)
**Bermudian**		
Yes	322 (81)	318 (80)
No	76 (19)	80 (20)
No answer	2 (0.5)	2 (0.5)
**Parish**		
Sandys/Southampton	72 (18)	72 (18)
Warwick/Paget	93 (23)	95 (24)
Pembroke/Devonshire	112 (28)	121 (30)
Hamilton/Smith’s/Saint George’s	115 (29)	104 (26)
No answer	8 (2)	8 (2)

Overall, 94% (*N* = 376) of survey participants were aware of the sugar tax implemented in Bermuda, while 32% (*N* = 128) were aware of the reduction in tax on select fruits and vegetables, Table 2. Most respondents (67%, *N* = 268) felt the tax was not an appropriate way to motivate people to adopt a healthier diet, Table 2. The main reasons cited for this view were the perceptions the tax would not be effective in changing behaviour (44%, *N* = 117) and the perceived high price of healthy food (20%, *N* = 53), Table 3. When asked how consumption of products containing added sugar had changed over the past two years, 48% (*N* = 192) reported they were now consuming fewer of these products, 44% (*N* = 176) indicated their consumption had stayed about the same, and 7% (*N* = 28) reported an increase in consumption (Additional File 1, Table S2). Households earning less than $150,000 annually (< $75,000 and $75,000–150,000 categories), and black residents were more likely to report consuming fewer products with added sugar (Additional File 1, Table S2).

**Table 2:** General population perspectives of the sugar tax in Bermuda, Bermuda Omnibus Survey, first quarter 2020

Perspectives	Yes % (n)	No % (n)	No answer % (n)
Awareness of sugar tax	94% (*N* = 376)	6% (*N* = 24)	
Awareness of fruit and vegetable tax reduction	32% (*N* = 128)	68% (*N* = 272)	
Acceptability of sugar tax	33% (*N* = 132)	67% (*N* = 268)	
Impact of sugar tax	48% (*N* = 192)	51% (*N* = 204)	1% (*N* = 4)

Responses indicate weighted percentage and sample of participants. *N* = 400. Awareness represents knowledge of sampled population towards the sugar tax or the tax reduction. Acceptability refers to a belief that the tax was an appropriate way to motivate people to adopt a healthier diet. Impact of the sugar tax refers to reduced consumption of products that contain added sugar compared to consumption two years prior

**Table 3:** Reasons cited for acceptability and consumption behaviours, Bermuda Omnibus Survey, first quarter 2020

	**Percentage (%), N**
**Reasons for non-acceptability of sugar tax (*N* = 267)**	
Tax won’t change people’s behaviour/people eat what they want	44%, *n* = 117
High price of healthy food/healthy food did not become cheaper	20%, *n* = 53
Education is more effective	13%, *n* = 35
Need to motivate people to eat healthier or lifestyle change	10%, *n* = 27
Personal choice or belief people’s food choices should not be dictated by taxes	6%, *n* = 16
Tax revenue for the government	4%, *n* = 11
Do not know/no answer	3%, *n* = 8
**Reasons for consuming more products with sugar (*N* = 28)**	
Healthy foods were less affordable	55%, *n* = 15
Healthy foods were less available	47%, *n* = 13
You are used to these products, and they are part of your diet	41%, *n* = 11
You do not always have access to drinking water	18%, *n* = 5
Some other reason	41%, *n* = 11
**Reasons for consuming fewer products with sugar (*N* = 183)**	
Trying to make diet healthier	93%, *n* = 170
Products with added sugar are becoming increasingly more expensive	43%, *n* = 79
Likes products less	34%, *n* = 62
Some other reason	18%, *n* = 33
**What has replaced products containing added sugar (*N* = 400)**	
Have not replaced products with added sugar with anything	47%, *n* = 188
Water	21%, *n* = 84
Fruits	14%, *n* = 56
Vegetables	6%, *n* = 24
Sugar substitutes	5%, *n* = 20
Honey	4%, *n* = 16
Sugar free/Low sugar products	3%, *n* = 12
More natural products	2%, *n* = 8
Do not know/no answer	3%, *n* = 12

Results represent percentage and number of participants that indicate key mentions from total unaided mentions. Participants could indicate multiple reasons. Subgroup comparisons were not provided due to small sample sizes. *N* = 400

Among the respondents who reported decreased consumption (*N* = 183), the primary reasons reported were to have a healthier diet (93%, *N* = 170) and because products with added sugar have become more expensive (43%, *N* = 79), Table 3. Among the respondents reporting increased consumption of sugary products 7% (*N* = 28), the main reasons reported were because healthy foods were less affordable and available, but also due to habitual dietary consumption of these products, and not having access to drinking water Table 3. Participants were also asked what, if anything, they had replaced products containing added sugar with. Nearly half of respondents (47%, *N* = 188) had not replaced these products with anything, whilst water was a replacement among 21% (*N* = 84) and fruit a replacement for 14% (*N* = 56), Table 3.

### Statistical analyses

Pearson’s chi-squared tests of independence demonstrated statistically significant relationships between awareness of the sugar tax and race [Χ^2^ (2, *N* = 369) 15.53, *p* =  < 0.001] and household income [Χ^2^ (2, *N* = 369) 13.00, *p* =  < 0.05]. Post hoc analysis of these results found that individuals who did not identify as either black or white race (“other” category) and lower income households (< $75,000) were less likely aware of the tax, Table 4. There was also a statistically significant relationship between awareness of the healthy food subsidy and sex [Χ^2^ (1, *N* = 400) 4.87 *p* =  < 0.05] and age [Χ^2^ (2, *N* = 400) 6.85, *p* =  < 0.05]. Males were more likely to be aware of the subsidy and younger adults (18–34 years) were less likely aware though these findings were no longer significant after correction for multiple comparisons. Finally, there was a statistically significant relationship of acceptability of the sugar tax and sex [Χ^2^ (1, *N* = 400) 5.02 *p* =  < 0.05] and household income [Χ^2^ (2, *N* = 369) 17.94, *p* =  < 0.001]. Males were more likely than females to believe the tax was an acceptable strategy to motivate healthier behaviours though this finding was no longer significant after correction for multiple comparisons. Middle income households ($75,000- < $150,000) were less likely to report the tax acceptable compared to lower (> $75,000) and high-income households (≥ $150,000). No other statistically significant differences were observed, Table 4.

**Table 4:** Relationship between sociodemographic variables and awareness and acceptability of the Bermuda sugar tax

	Awareness of the sugar tax			Awareness of the fruit and vegetable subsidy			Acceptability of the sugar tax		
	Yes (n)	No (n)	*P*-value	Yes (n)	No (n)	*P*-value	Yes (n)	No (n)	*P*-value
Sex			0.427			*p* < .05			*p* < .05
Female	214	10		62	162		64	160	
Male	165	11		67	109		69	107	
Age			0.93			*p* < .05			0.692
18–34	58	7		12	53		22	43	
35–54	160	7		57	110		59	108	
55 +	161	7		60	108		52	116	
Race			*p* < .001			0.829			0.604
Black	167	10		57	120		54	123	
White	150	3		51	102		54	99	
Other	32	7		11	28		14	25	
Household income			*p* < .05			0.729			*p* < .001
< $75,000	116	14		89	41		52	78	
$75- < $150 K	130	3		91	42		26	107	
$150 K +	104	2		68	38		45	61	

Non-responses removed from analysis; *K* Thousand, *n* Number

### Key informant interview results

There were 16 key informants contacted to participate in the study, of which 14 agreed to participate (88% response rate). Of the 14 participants, four were from the government sector, four were from the health sector, and six were from the food and beverage sector (Table 5).

**Table 5:** Stakeholder categories: Key informant interviews, Bermuda (*N* = 14)

Sector	Description	Number
Government	Civil servants, public officers, members of Parliament (including Opposition)	4
Health	Health practitioners, nutritionists, health promotion advocates	4
Food and Beverage	Restaurants, grocery stores, wholesalers, convenience stores, beverage distributors	6

The key themes, sub-themes and associated beliefs are presented in turn with illustrative quotes included in Table 6. The full results by theme and sub-theme are included in Additional File 1.

### Awareness

#### Knowledge and understanding of the tax

All informants (*N* = 14) were aware of the sugar tax. Government and health sector participants explained the substantial local media attention contributed to high awareness of the tax (Table 6). Just over half of informants (*N* = 8) reported the stated aim of the sugar tax was to motivate people to make healthier choices. However, informants from all sectors (*N* = 7) questioned the true motivations for the tax and whether the primary aim was to generate revenue for the government (Table 6). This perspective was countered by health and government sector informants (*N* = 3) who expressed the tax was multi-pronged, targeting both health and financial aspects (Table 6).

Despite high general awareness, informants expressed a more limited understanding of the tax policy, notably the items subject to the tax. All informants were aware that sugar-sweetened beverages were included in the tax, but a lack of clarity was reported regarding the tax on sugar-sweetened food products and the duty reduction on select healthy food items (Table 6). While most informants (*N* = 10) reported some form of general awareness of this duty reduction, only those directly involved in the tax implementation had knowledge of the healthy foods receiving the duty reduction.

### Beliefs about appropriateness and acceptability

#### Taxation as a strategy for health promotion

Perceptions on the appropriateness of using taxes as a strategy for health promotion varied across informants, including within each sector. Overall, most informants (*N* = 10) reported some level of support for taxation to address health issues. Participants cited the high level of diabetes, overweight, and obesity in Bermuda and the associated unsustainable health-related costs. However, this support was conditional on policy implementation, the use of revenue, and the resulting behavioural and health outcomes (Table 6). Strong support for the sugar tax in Bermuda was expressed from a government informant who reflected *“we definitely think it is the right approach”* (P09 Government). Informants in strong opposition expressed the belief it was not appropriate because sugar taxes *“don’t work*” (P06 Food and Beverage) to effect behaviour change that will result in improved health outcomes. Informants opposed also voiced the right to choose what to consume without being penalised (Table 6).

Most informants (*N* = 8) expressed beliefs the sugar tax as implemented in Bermuda was not an appropriate example of an intervention implementation to address health issues. Highly cited factors affecting perceptions of the tax as implemented included the absence of accompanying education, health promotion, or new health initiatives, and the lack of transparency surrounding the use of sugar tax revenue (Table 6).

#### Tax mechanism and level

There were mixed responses surrounding the appropriateness of the tax mechanism, an import tariff levied by Customs. The use of an import tariff was viewed as *“the easiest way to do it”* (P06 Food and Beverage) from a feasibility perspective and described by a government sector informant as the pragmatic approach (Table 6). However, informants questioned the appropriateness of administering the tax using import tariffs, indicating it allowed for subsequent variability in the pass-through of the tax to the consumer and did not result in a clear price differentiation at the retail level (Table 6). Informants from all sectors suggested a tax administered at point-of-sale would more appropriately reflect price differentiation and make sugar-taxed products clearer to consumers (Table 6). Informants felt the level of tax – a 75% duty on the import price of a product – was high and could create challenges for the food and beverage industry to apply and still be able to sell products. It was reported the 75% duty was much higher than all other duties on food, one of the highest import duties in Bermuda, and one of the highest sugar taxes in the world (Table 6). However, it was also believed that while the level of tax seems high, the effect at the retail level was expected to be much lower, *“because a lot of the price on the shelf is markup…The price on the shelf would have not been going up that much”* (P14 Government).

#### Intervention targets

Informants from all sectors questioned the appropriateness of the items subject to the sugar tax and the consideration given to determining these products (Table 6). Participants from both the food and beverage and health sectors expressed frustration the amount of sugar in a product was not a factor in it was subject to the tax (Table 6). Food and beverage sector informants further questioned the appropriateness of including what they felt were “healthier” products in the tax (Table 6). Two health sector informants felt a sugar-sweetened beverage tax would have been more appropriate as it would have been simpler, supported clearer messaging, facilitated measurable outcomes, and would have aligned with sugar taxes implemented. The expansion beyond taxing sugar-sweetened beverages in Bermuda was perceived by health sector informants (*N* = 2) to have *“diluted the message*” (P02 Health).

### Beliefs about economic and equity impacts

#### Impact on prices

All informants reported the sugar tax had an impact on product prices, but a range of beliefs were expressed. Food and beverage sector informants reported that certain items *“have become incredibly more expensive”* (P13 Food and Beverage) due to the tax. Informants from all sectors felt that at the consumer level, the impact of the sugar tax in terms of creating a price differentiation between sugar-taxed and non-sugar taxed was often not clearly observed Table 6). Nearly all informants from the government and health sectors (*N* = 6) felt that all prices – taxed and non-taxed products – have increased (Table 6). Informants expressed the belief that some of tax burden was spread to non-taxed products, while others felt the sugar tax presented an opportunity for importers and retailers to raise prices (Table 6). Government and health sector informants also questioned whether full pass-through of the sugar tax was even feasible if it resulted in a final retail price deemed “unsellable”. Two food and beverage sector informants confirmed some tax absorption occurred for this reason.

#### Affordability of healthy food

Informants from all sectors agreed that healthy food is expensive in Bermuda, but also reported that all food is expensive and *“everything on this island, it’s expensive”* (P08 Health). Informants suggested the price of healthy food may simply be too high to motivate behaviour change towards healthier eating (Table 6). Food and beverage informants reported the sugar tax has increased the cost of what may be considered healthier substitutions, as products that are low in sugar are still included in the tax (Table 6).

#### Impact on socioeconomic equality

Informants from all sectors reported the sugar tax increases the cost of living because “*it makes everything more expensive* “ (P06 Food and beverage), and felt that those with limited incomes would be most affected (Table 6). A health sector informant explicitly expressed the concern that the sugar tax as implemented in Bermuda “*has become rather more of* a *regressive tax”* (P03 Health). In contrast, one health sector participant disagreed with this argument, stating that all individuals can choose whether to consume sugar-taxed products and thus can choose whether they are financially affected by the tax.

#### Use of tax revenues

No informants were aware of any direct use of revenue generated from the sugar tax. Nearly all informants from the food and beverage and health sectors (*N* = 8) expressed concerns the revenue raised was *“just going into the general government coffers”* (P01 Food and Beverage) as opposed to being used to fund health education, new health programs or public health strategies to combat obesity and chronic disease. It was confirmed by informants from the government sector that sugar tax revenue was not ring-fenced and instead was pooled with all other tax revenue. It was explained *“there was a change in mindset”* (P14 Government) during the implementation process and decided that sugar tax revenue would not specifically be used to fund health initiatives but was instead *“just tax revenue”* (P09 Government).

### Beliefs about the effectiveness of the tax as a health promotion measure

#### Impact on purchasing and consumption of sugar-taxed products

Most informants felt the sugar tax has not substantially affected purchasing and consumption of sugar-taxed products, but responses were mixed. Government sector informants (*N* = 2) reported some reduced consumption of sugar-sweetened beverages but also indicated this behaviour change may have been temporary with consumers now accustomed to the increased prices (Table 6). All food and beverage sector informants reported they have not observed any substantial or sustained changes and instead that purchases of sugar-taxed items have “*stayed pretty consistent”* (P05 Food and Beverage). It was further emphasised (*N* = 3) that there have been natural declines in soda consumption and increased consumption of healthier alternatives, trends that have been happening for many years prior to the tax (Table 6). It was also articulated that behaviour change was unlikely unless there was a more substantial price differential observed at the retail level.

#### Impact on health outcomes

None of the informants were aware of an impact on health outcomes as a result of the sugar tax. It was acknowledged it was likely too soon to observe health outcomes. However, informants from all sectors questioned whether there would, or could, be any beneficial health outcomes without the accompaniment of health and education programmes (Table 6). Health sector participants (*N* = 2) expressed the sugar tax was a *“missed opportunity”* to establish education and healthy eating campaigns in schools and the community and to garner public support which could lead to behaviour change and improved health outcomes. Participants from all sectors expressed the belief that given the design and implementation of the tax policy, it would be unlikely to result in public health impact (Table 6).

#### Impact of the healthy food subsidy

Among informants aware of the elimination of import duty on select fruits and vegetables (*N* = 10), all felt the reduction from 5 to 0% was too small to affect retail prices, and thus consumption. Food and beverage and health sector informants added that most products included in the subsidy were grown locally and often embargoed from import further affecting any potential impact (Table 6). Food and beverage informants indicated the high retail costs of imported fruits and vegetables are predominantly driven by overseas availability, seasonality, and fuel prices and expressed there are *“way too many factors for the 5% duty drop to make a lot of difference”* (P06 Food and Beverage). A government informant described the reduction in import duty on select produce items as a *“give and take thing from the government”* (P04 Government). Similarly, food and beverage and health sector informants perceived the duty reduction as a symbolic gesture given the minor tax reduction and the inclusion of products with seasonal import embargoes.

**Table 6:** Themes, sub-themes, and illustrative quotes from key stakeholder interviews

Themes and sub-themes	Illustrative quotes
**Awareness**	
Knowledge and understanding of the tax	*“Doing something as drastic as the sugar tax was, it did generate a lot of conversation in Bermuda.”* (P03 Health) *“The aim was to make more money and dress it up as something it is not.”* (P13 Food and Beverage) *“One [aim} was to create a dialogue around healthy eating… The second [aim} was to actually raise some money that we were going to use for various health initiatives.”* (P14 Government) *“I don't think [the tax] is clear, completely, to me. And I certainly don't think it's clear to the public.”* (P02 Health)
**Beliefs about appropriateness and acceptability**	
Taxation as a strategy for health promotion	*“I think in some cases [sugar taxes] are a useful design and they can have a positive impact on our health as a population strategy. Yes, so I am in favour of them, [when rolled] out the right way, and in a way that's meaningful, and a way that gives back into programs that will further improve public health.”* (P03 Health) *I'm 100% in favour of health regimes. I'm 100% in favour of control of obesity… By the same token, I also respect people's ability to choose.”* (P11 Government) *I think it needs to be very clear where that money is being put afterwards and to what it has been put to use for. Then I think it's very acceptable. But this is not the case.”* (P13 Food and Beverage)
Tax mechanism and level	*“We decided quickly that we weren't going to be able to [administer the tax] at point-of-sale because we don't have any tax structure that would allow that. And it would also, if we did put in infrastructure to facilitate that, it would wipe out any benefit of that the tax. It would be very expensive to implement.”* (P14 Government) *“So the wholesalers import the goods and pay the 75%. And then they sell it to the grocery stores. And then the grocery stores sell it to [the consumer]. So then you have got all the different mark-ups or markdowns.”* (P04 Government) *“There's no point-of-sale effect that you can say, ‘Oh, you know what, that's cheaper’. So I know I should have the diet or the sugar free one, so let me just go buy that because it's cheaper.”* (P03 Health) “*The import duty on a sugary drink, I think is more than cigarettes, more than tobacco.”* (P06 Food and Beverage) *“I think we're the only country in the world that has a rate this high*.*”* (P04 Government)
Intervention targets	*“So, the question begged as to how carefully the implementation and the determination as to what categories would be covered. I don't believe that was well thought through.” (*P11 Government) *“…what was so confusing, or frustrating, was that it didn't matter if it was 50 g of sugar per serving, or 0.5 g of sugar per serving.”* (P07 Food and Beverage) *“…we were having items dutied that in our opinion had nothing to do with what the sugar tax should have been focusing on. Things like protein powders and smoothies were being hit with the tax.”* (P01 Food and Beverage)
**Beliefs about economic and equity impacts**	
Impact on prices	*“By the time the consumer goes to the shelves…the way that the cost was spread by the wholesalers and the retailers, creates virtually no price differentiation. In some instances, there is zero price differentiation between a diet soda and a regular soda.”* (P09 Government) *“… the sugar tax, it just made the price of all foods go up. That's my personal experience and I've heard other people say the same thing.”* (P08 Health) *“But that food right beside the one that was taxed when it landed, has gone up to absorb the price of the one that was taxed at 75%. So they have all gone up.”* (P03 Health) *“Sugar or no sugar, this seems to be the excuse to be able to increase costs, across the board.”* (P11 Government)
Affordability of healthy food	*“If a bag of apples is still going to be $9, then a Snickers bar at $1.85 is still going to be less expensive and may go into a kid’s lunchbox instead.”* (P01 Food and Beverage) *“[Drinks that] have less than 1% sugar in them, but they’re still hit with the sugar tax. So at the end of the day if you really look at trying to push somebody from a [high-sugar soda] to a one gram [of sugar flavoured sparkling water], it’s very difficult to do that when they’re both hit with the same tax.”* (P13 Food and Beverage)
Impact on socioeconomic equality	*“…all you're going to do by increasing the duty on [sugar-taxed items], is you're going to increase the ultimate price. And that's going to disadvantage the poorer consumer. Because people who only have a certain amount of disposable income, spend a disproportionately large amount on food and beverage.”* (P07 Food and Beverage)
Use of tax revenues	*“The $1 million, $3 million that [the government] grossed in the first year [did not come] back directly into any specific new [health education] curriculum.”* (P03 Health) *“Well what the government, and the Premier specifically, committed to was that all of [sugar tax revenue] would be used for health initiatives. Or health promoting initiatives. Or initiatives that would advance health… That side of things has not panned out quite as intended.”* (P09 Government)
**Beliefs about the effectiveness of the tax as a health promotion measure**	
Impact on purchasing and consumption of sugar-taxed products	*“We did observe through surveys that consumption of sugary items, of sodas in particular for which we had benchmark data, had gone down.”* (P09 Government) *“People have just gotten used to paying whatever they’re paying for their sweet drink. Yeah, I’m not sure it has actually caused any lasting behaviour change.”* (P14 Government) *“It’s naive to say that the implementation of the sugar tax has had a massive benefit because people buy less sugary sodas. That’s been on the decline for 10 years.”* (P01 Food and Beverage*)* *“Nobody is going to tell me that if you have to pay more for your soda, and you pay more for your candies, that you’re going to forego those choices and buy some carrots.”* (P11 Government)
Impact on health outcomes	*“No, I don’t think it will [have an impact on health] …it’s not a sustainable change for changing people’s behaviour around chronic disease risk factors.”* (P14 Government) *“If we had specifically designed programmes and further education that were as a result of the revenue from [the tax], then perhaps we'll see an effect of that in improving health.* (P03 Health) *“It was not well thought through, not well implemented; and therefore, it's our opinion that it has none of the advertised effect on the public health that it was supposed to.”* (P12 Food and Beverage)
Impact of the healthy food subsidy	*“…when [the government says] they have reduced the duties on cauliflower or broccoli… it literally amounts to three or four cents a pound. Half of the products that they talk about are embargoed six to eight months a year. So there is no impact at all.”* (P01 Food and Beverage)

Bold font denotes themes, regular font is used for sub-themes, and italics for illustrative quotes

## Discussion

To our knowledge, this is the first study to investigate perceptions of the Bermuda sugar tax. The findings from the survey of the general population indicate a high level of awareness of the tax which was greater than awareness for similar taxes implemented in other settings including Mexico (46–65%) [17, 18], Barcelona, Spain (83%) [19], the United Kingdom (68–92%) [18, 20], and Berkeley (52–70%), Oakland (39%) and San Francisco (30%) in California, United States [21]. Key informants substantiate this finding adding that substantial local media attention and discussions in the community contributed to widespread awareness of the tax. This finding is important because evidence suggests tax awareness through media coverage and campaigns, and political dialogue surrounding the tax, can impact behaviour change irrespective of price increases [22, 23]. Awareness varied by race and income, but did not vary by age or sex, with less awareness among minority populations and lower income households which may be due to differing exposure to media or government messaging. Disparities in awareness of health taxes can affect success by reducing consumer response to the tax. Targeted education campaigns would be beneficial to address these disparities. There was also limited knowledge of the healthy food subsidy which may limit its effectiveness to motivate healthier choices.

How a tax is implemented can affect its acceptability [24]. A pre-implementation survey found more support than opposition for a sugar tax in Bermuda [10], but the current study found the opposite after policy implementation. Acceptability was higher among males, but most of the general population felt taxation was not an appropriate strategy to motivate healthier dietary choices, primarily because they believed taxes do not work to change behaviour. This sentiment was reiterated by key informants; however, most did still express support for a well-designed policy implemented in a way that results in beneficial health outcomes, and where the revenue generated was used for new health initiatives. Carefully-designed, comprehensive policies that include complementary actions to promote and support healthier diets are necessary to achieve improved population health [25]. The Bermuda sugar tax was implemented without accompanying health initiatives, health education or health promotion campaigns which was identified as a missed opportunity that affected acceptability of the tax.

Acceptability of health taxes is also influenced by how tax revenue is used [26]. Greater public acceptability of sugar sweetened beverage taxes has been reported when revenue generated is used for public health [27, 28], or when there is transparency between taxes and the purposes they intend to serve [29]. Health taxes without transparency of the primary goal are particularly vulnerable to criticism [30]. The Bermuda sugar tax has been very successful in generating revenue – $US 4.7 million in 2019 which is approximately ten times the amount raised by the UK Sugar Drinks Industry Levy as a proportion of total tax revenues [31, 32]. However, despite being promoted as a public health measure, the revenue raised from the Bermuda sugar tax contributed to the general tax fund without earmarking for health. Informants were unaware of use of tax revenue for health purposes, or new health initiatives born out of the revenue generated. This affected perceptions of the tax with informants questioning whether the sugar tax was primarily financially motivated but promoted as a public health initiative. The intended use of tax revenue reportedly changed during policy implementation, going from use for specific health initiatives to use as general tax revenue. These findings are similar to experiences in South Africa where the true motivation of a sugar-sweetened beverage tax was also questioned [33], and French Polynesia where soft drink tax revenue was redirected as government priorities changed [34]. Countries designing similar policies should consider that public support for a new tax can be boosted if there is a strong link between the tax aim and use of revenue. Soft earmarking of tax revenue is a recommended strategy to achieve this [35].

The mechanism used to administer health taxes can also play an important role in both public perceptions and impact of the tax. Use of existing taxation mechanisms has been a key factor for success in other settings [34]. In Bermuda, the use of import tariffs and an *ad valorem* tax structure was the most feasible mechanism for implementation and practical for an island setting heavily reliant on imports. However, this came at the expense of clear price signalling of taxed products to consumers as much of the final retail price is determined by other costs (e.g. shipping, distribution, retail costs) which are not taxed. *Ad valorem* tax structures, as opposed to specific taxes, also incentivise trading down to cheaper brands which may be even less healthier [36]. To support acceptability and potential impact, there is a need for careful design of the tax with consideration for the type of tax applied, the context in which the tax is applied and the tax objective.

While most general population respondents reported the belief the sugar tax would not have an impact on behaviour, participants did report decreased consumption of products containing added sugar, in part due to price increases. Lower income households and black residents were more likely to report reduced consumption. Key stakeholders similarly felt the tax would not have a lasting impact on behaviour but did report some short-term changes in consumption were observed. Quantitative evaluation of the tax found that consumption of sugar-sweetened beverages decreased following implementation of the tax [37]. This suggests an inconsistency between perceptions and behaviour, a finding supported by previous research [38].

To support individuals to make healthier dietary choices, healthy foods need to be available and affordable. Healthy food was reported as expensive by both the general population and key informants in Bermuda, and the minor tax reduction on select produce was not believed to have an impact on retail prices. This perception was consistent with the findings from quantitative evaluation of the tax [37] and from the experience of a similar subsidy implemented in Tonga [39], and further supports guidance from the World Health Organization that a 10–30% reduction in healthy food prices is needed to incentivise increased consumption [40].

There are several unique features of the country characteristics and design of the sugar tax in Bermuda compared to similar taxes implemented in other countries. First, there was no concerted industry effort to block the tax as has been observed in other settings [41, 42]. With no major local food or beverage producers, industry did not exhibit the power and influence on health taxes as observed in the United States and elsewhere [43, 44]. Second, as an island setting with nearly all food and beverages imported, the sugar tax in Bermuda is difficult to avoid. Retailers are reliant on imported food and the island setting provides substantial barriers to cross-border shopping by consumers. Third, with no local sales tax, an import tariff structure already established, and a heavy reliance on imported food, the use of import tariffs to administer the tax was the most practical and feasible option in this setting. Finally, with limited local production, a tax design that promoted product reformulation was not relevant in this setting but may have high relevance in other settings as observed with the United Kingdom Soft Drinks Industry Levy [45]. The reflections presented in this study should be interpreted with consideration of these factors.

### Limitations

This study aimed to provide a broad exploration of perceptions of the sugar tax in Bermuda. The data obtained from the Bermuda Omnibus Survey and the key informant interviews are self-reported and subject to both recall and social desirability response biases. Literature on tax compliance has illustrated self-reported behaviour can differ from actual behaviour [46] and analyses of sugar-sweetened beverage taxes have shown how perceptions on the impact of these taxes can be inconsistent with scientific literature [38]. There may also be natural changes in consumption behaviour over time, which was noted by both study populations. The survey findings should be interpreted in this context, particularly given the inconsistencies between perceptions of the impact of the tax in Bermuda and reported consumption patterns and consumer sales data [37]. Further, the survey data represent views at a single point in time. There was no baseline measure to formally assess changes in measures before and after the sugar tax implementation. Data from the Sugar Tax Consultation period were consulted to provide a general indication of pre-survey beliefs but were not a representative sample. It is possible that other public policies implemented around the same time as the tax could bias perceptions of the sugar tax implementation and its impact.

The perspectives from the key informant interviews reflect individual perspectives across different sectors; these findings are not generalisable to all stakeholders in the sector, nor are they generalisable more broadly in the community. The authors’ interpretations of the findings are subject to personal and professional biases. Potential for biases were mitigated by purposively sampling a wide range of informant perspectives, cross-referencing emerging findings in subsequent interviews and with public documents, and conducting coding comparisons. Despite the limitations, it is still possible to compare the findings from this study with the findings in similar and different settings. The results reflect general agreement between the two study populations, and they align with findings from health tax evaluations in other settings which lends further validity to the study findings. The use of qualitative methods allowed for the collection of in-depth and detailed responses providing a rich data source to understand beliefs surrounding awareness, acceptability and perceived impact of the tax implemented in this setting. Other settings can draw on these findings in the design of similar polices but should consider the local context and perceptions.

## Conclusions

There was high awareness of the sugar tax in Bermuda, but limited acceptability of the tax as implemented. Acceptance and support of a sugar tax in this setting appeared conditional on tax implementation. Clarity in the tax policy, appropriateness of the tax mechanism, and use of revenue in alignment with the tax aim are key components to achieve public and key stakeholder acceptance. The use of economic tools to incentivise consumption behaviours is a policy intervention recommended to promote healthy diets and reduce obesity. However, motivating dietary change will require comprehensive tax policies which incorporate education and health promotion and support healthy food environments, including the increased availability and affordability of healthy substitutions. The Bermuda sugar tax was implemented without these complementary components which may limit the potential health impacts of the tax. The experience and lessons learned in Bermuda can inform the development and implementation of similar policies in other settings.

## Supplementary Information

**Additional file 1.**

## Data Availability

The datasets generated during the current study are not publicly available due to limitations of ethical approval involving the patient data and anonymity but are available from the corresponding author on reasonable request.

## Abbreviation

OECD: Organisation for Economic Co-operation and Development
