# Supplementary material for: The ‘sugar tax’ in Bermuda: a mixed methods study of general population and key stakeholder perceptions
Source: BMC Public Health. 2022 Aug 16;22:1557. doi: 10.1186/s12889-022-13945-9 (PMC9379233; doi:10.1186/s12889-022-13945-9)
Supplement: Supplementary file 1 — Additional file 1. [file 12889_2022_13945_MOESM1_ESM.docx]

# Additional File 1

## Bermuda Omnibus Survey Commissioned Questions

I1. Thinking back over the past 2 years, would you say that your consumption of products with added sugar, such as soft drinks or desserts and snacks, is now **[READ RESPONSES IN ORDER]**than it was two years ago? **CODE ONE RESPONSE ONLY**

1. Much more
2. Slightly more
3. About the same amount
4. Slightly less, OR
5. Much less

VOLUNTEERED

98Don’t know/No answer

I2. **[POSE IF CODES 3 OR 4 IN PREVIOUS]:**Why has your consumption of products with added sugar decreased? **[POSE IF CODE 1 IN PREVIOUS]:**Why has your consumption of products with added sugar increased? **[POSE IF CODE 2]:**Why has your consumption of products with added sugar stayed the same? **CODE ALL THAT APPLY; DO NOT READ**

PRECODES IF CODES 1 OR 2 IN PREVIOUS

1. These products are more affordable/Healthy foods (i.e., fruit, vegetables, whole grain products) are less affordable
2. Healthy foods (i.e., fruit, vegetables, whole grain products) are not available or easily available
3. These products are part of my diet and I am used to them
4. I don’t always have access to drinking water
5. I only consume a small amount of these products
6. Other (please specify: ________)
7. Don’t Know/No Answer

PRECODES IF CODE 3 IN I1

1. Products are becoming increasingly expensive/Cost
2. Because they are unhealthy/Focusing on my health
3. I like them less than before
4. Other (please specify: ____________)

98Don’t know/No answer

I3.**ASK ALL]**What, if anything, have you replaced products containing added sugar with? **[IF NEEDED:**Products with added sugar could include, for example, soft drinks or desserts and snacks**]**

**RECORD VERBATIM RESPONSE**___________________________________________

96Have not replaced products with added sugar with anything

98Don’t Know/No Answer

I4. **[ASK ALL]**Were you aware that in 2019, a tax was applied to food and beverages with added sugar because of concerns about their effects on people’s health? **CODE ONE ONLY**

1. Yes
2. No

I*5.* **[ASK ALL]**Were you aware that in 2019, taxes applied to fruits and vegetables were reduced because they are healthy foods? **CODE ONE ONLY**

1. Yes
2. No

I6.**ASK ALL]**In your opinion, is the tax on food or drinks with added sugar an appropriate way to motivate people to have a healthy diet?**CODE ONE ONLY**

1. Yes
2. No (why not, please, specify: _____________)?

## Key informant semi-structured interview schedules

**Sector: Food and beverage**

**Introduction**

(re-state while recording that informed consent received, permission to record received)

1. Can you please provide a brief overview of your background?

**Awareness**

1. Could you please outline the extent of your knowledge of the sugar tax in Bermuda?
2. Is it clear to you which items are included in the tax?

*Prompt: Ask participant to describe their understanding of the tax*

1. Are you aware of any subsidies on healthy food items as part of the Sugar Tax implementation?

*Prompt: Ask participant to describe their understanding of subsidised* items

1. What is your understanding of the aim of the sugar tax in Bermuda?
2. What is your understanding of how revenue from the sugar tax will be used?
3. Were <*you/your organization/company>* involved in the development or consultation process for the sugar tax? If so, how?
4. How does this tax apply to your business?

**Acceptability**

1. Thinking from your perspective as *<insert position here>*how acceptable is the Sugar Tax in Bermuda and why?

*Prompt: How acceptable is the level of the tax (75%)? The products taxed? The use of food/beverage taxes to address health issues (obesity, NCDs)*

1. What do you see as the benefits of this tax?

*Prompt: Are there factors that would facilitate the acceptability of this tax?*

1. What concerns do you have about this tax?

*Prompt: what factors inhibit the acceptability of this tax?*

1. Who is this tax good for? And perhaps not-so-good for?

**Impact**

1. How does this tax affect your business? And your customers?
2. Have your business practices changed since the implementation of this tax? If so, how?

*Prompt: Changes in purchasing, prices*

1. Have your customers/clients changed their habits since the implementation of the tax? If so, how?

*Prompt: Changes in purchasing?*

**Closing**

1. Are there any other points that you would like to raise regarding the sugar tax in Bermuda?
2. Can you suggest other key stakeholders we should speak with?
3. Do you have any questions or any final comments?

**Sector: Government; health**

**Introduction**

(re-state while recording that informed consent received, permission to record received)

1. Can you please provide a brief overview of your background?

**Awareness**

1. Could you please outline the extent of your knowledge of the sugar tax in Bermuda?
2. Is it clear to you which items are included in the tax?

*Prompt: Ask participant to describe their understanding of the tax*

1. Are you aware of any subsidies on healthy food items as part of the Sugar Tax implementation?

*Prompt: Ask participant to describe their understanding of subsidised items*

1. What is your understanding of the aim of the sugar tax in Bermuda?
2. What is your understanding of how revenue from the sugar tax will be used?
3. Were <*you/your organization/company>* involved in the development or consultation process for the sugar tax? If so, how?

**Acceptability**

1. Thinking from your perspective as *<insert position here>*how acceptable is the Sugar Tax in Bermuda and why?

*Further probe: How acceptable is the level of the tax (75%)? The products taxed? The use of food/bev taxes to address health issues (obesity, NCDs)*

1. What do you see as the benefits of this tax?

*Further probe: Are there factors that would facilitate the acceptability of this tax?*

1. What concerns do you have about this tax?

*Further probe: what factors inhibit the acceptability of this tax?*

1. Who is this tax good for? And perhaps not-so-good for?

**Impact**

1. It is now one year on from the implementation of the full Sugar Tax – In your opinion, what has been the impact(s) of this tax?

*Prompts: changes in prices, availability of SSB/sugary foods, consumption, health*

**Closing**

1. Are there any other points that you would like to raise regarding the sugar tax in Bermuda?
2. Can you suggest other key stakeholders we should speak with?

## Standards for Reporting Qualitative Research

|  | **Standards for Reporting Qualitative Research (SRQR)*** |  |
| --- | --- | --- |
|  | <http://www.equator-network.org/reporting-guidelines/srqr/> |  |
|  |  |  |
|  | **Table S1. Standards for Reporting Qualitative Research** |  |
| **Title and abstract** | | **Page/line no(s).** |
|  | **Title** - Concise description of the nature and topic of the study Identifying the study as qualitative or indicating the approach (e.g., ethnography, grounded theory) or data collection methods (e.g., interview, focus group) is recommended | Page 1 |
|  | **Abstract** - Summary of key elements of the study using the abstract format of the intended publication; typically includes background, purpose, methods, results, and conclusions | Page 2 |
|  |  |  |
| **Introduction** | |  |
|  | **Problem formulation** - Description and significance of the problem/phenomenon studied; review of relevant theory and empirical work; problem statement | Page 4-5 |
|  | **Purpose or research questio**n - Purpose of the study and specific objectives or questions | Lines 91-94 |
|  |  |  |
| **Methods** | |  |
|  | **Qualitative approach and research paradigm** - Qualitative approach (e.g., ethnography, grounded theory, case study, phenomenology, narrative research) and guiding theory if appropriate; identifying the research paradigm (e.g., postpositivist, constructivist/ interpretivist) is also recommended; rationale** | Page 6-7 |
|  | **Researcher characteristics and reflexivity** - Researchers’ characteristics that may influence the research, including personal attributes, qualifications/experience, relationship with participants, assumptions, and/or presuppositions; potential or actual interaction between researchers’ characteristics and the research questions, approach, methods, results, and/or transferability | Lines 169-174 |
|  | **Context** - Setting/site and salient contextual factors; rationale** | Page 6 |
|  | **Sampling strategy** - How and why research participants, documents, or events were selected; criteria for deciding when no further sampling was necessary (e.g., sampling saturation); rationale** | Page -6 |
|  | **Ethical issues pertaining to human subjects** - Documentation of approval by an appropriate ethics review board and participant consent, or explanation for lack thereof; other confidentiality and data security issues | Lines 138-139;  Lines 142-144  Lines 175-179;  Lines 519-523 |
|  | **Data collection methods** - Types of data collected; details of data collection procedures including (as appropriate) start and stop dates of data collection and analysis, iterative process, triangulation of sources/methods, and modification of procedures in response to evolving study findings; rationale** | Pages 6-7 |
|  | **Data collection instruments and technologies** - Description of instruments (e.g., interview guides, questionnaires) and devices (e.g., audio recorders) used for data collection; if/how the instrument(s) changed over the course of the study | Pages 6-7 |
|  | **Units of study** - Number and relevant characteristics of participants, documents, or events included in the study; level of participation (could be reported in results) | Lines 246-248; Table 5 |
|  | **Data processing** - Methods for processing data prior to and during analysis, including transcription, data entry, data management and security, verification of data integrity, data coding, and anonymization/de-identification of excerpts | Pages 6-7 |
|  | **Data analysis** - Process by which inferences, themes, etc., were identified and developed, including the researchers involved in data analysis; usually references a specific paradigm or approach; rationale** | Pages 6-7 |
|  | **Techniques to enhance trustworthiness** - Techniques to enhance trustworthiness and credibility of data analysis (e.g., member checking, audit trail, triangulation); rationale** | Pages 6-7 |
|  |  |  |
| **Results/findings** | |  |
|  | **Synthesis and interpretation** - Main findings (e.g., interpretations, inferences, and themes); might include development of a theory or model, or integration with prior research or theory | Pages 12-16 |
|  | **Links to empirical data** - Evidence (e.g., quotes, field notes, text excerpts, photographs) to substantiate analytic findings | Table 6; Additional File 1 pages 9-20 |
|  |  |  |
| **Discussion** | |  |
|  | **Integration with prior work, implications, transferability, and contribution(s) to the field -** Short summary of main findings; explanation of how findings and conclusions connect to, support, elaborate on, or challenge conclusions of earlier scholarship; discussion of scope of application/generalizability; identification of unique contribution(s) to scholarship in a discipline or field | Pages 16-18 |
|  | **Limitations** - Trustworthiness and limitations of findings | Page 19 |
|  |  |  |
| **Other** | |  |
|  | **Conflicts of interest** - Potential sources of influence or perceived influence on study conduct and conclusions; how these were managed | Lines 492-495 Lines 530-532 |
|  | **Funding** - Sources of funding and other support; role of funders in data collection, interpretation, and reporting | Lines 532- 544 |
|  |  |  |
|  | *The authors created the SRQR by searching the literature to identify guidelines, reporting standards, and critical appraisal criteria for qualitative research; reviewing the reference lists of retrieved sources; and contacting experts to gain feedback. The SRQR aims to improve the transparency of all aspects of qualitative research by providing clear standards for reporting qualitative research. |  |
|  |  |  |
|  | **The rationale should briefly discuss the justification for choosing that theory, approach, method, or technique rather than other options available, the assumptions and limitations implicit in those choices, and how those choices influence study conclusions and transferability. As appropriate, the rationale for several items might be discussed together. |  |
|  |  |  |
|  | **Reference:** |  |
|  | O'Brien BC, Harris IB, Beckman TJ, Reed DA, Cook DA. **Standards for reporting qualitative research: a synthesis of recommendations.** *Academic Medicine*, Vol. 89, No. 9 / Sept 2014  DOI: 10.1097/ACM.0000000000000388 |  |
|  |  |  |
|  |  |  |

## Bermuda Omnibus Survey, additional results

**Table S2. Consumption of foods and beverage with added sugar in Bermuda**

|  | Bermuda overall % | | Sex (%) | | Household Income (%) | | | | Age (%) | | | | | Race (%) | | |  |
| --- | --- | --- | --- | --- | --- | --- | --- | --- | --- | --- | --- | --- | --- | --- | --- | --- | --- |
|  |  |  | M | F | | <$75K | $75 -  <$150K | ≥$150K | | 18-34 | 35-54 | 55+ | Black | | White | Other | |
| Much more | | 3 | 4 | 3 | | 8 | 1 | 1 | | 2 | 3 | 4 | 4 | | 1 | 5 | |
| Slightly more | | 4 | 3 | 5 | | 3 | 3 | 6 | | 2 | 7 | 1 | 5 | | 2 | 5 | |
| About the same amount | | 44 | 47 | 42 | | 33 | 42 | 58 | | 46 | 44 | 43 | 33 | | 63 | 45 | |
| Slightly less | | 24 | 23 | 24 | | 30 | 27 | 14 | | 26 | 23 | 23 | 26 | | 20 | 23 | |
| Much less | | 24 | 24 | 25 | | 26 | 28 | 19 | | 25 | 21 | 28 | 32 | | 13 | 22 | |
| Don’t know  Not applicable/I don’t eat products with added sugar | | 0  1 | 0  1 | 0  0 | | 0  0 | 0  0 | 0  2 | | 0  0 | 0  0 | 0  1 | 0  0 | | 0  1 | 0  0 | |
| *Weighted sample size (#)* | | *400* | *190* | *210* | | *140* | *134* | *97* | | *91* | *176* | *133* | *198* | | *118* | *53* | |
| *Unweighted sample size (#)* | | *400* | *176* | *224* | | *130* | *133* | *106* | | *65* | *167* | *168* | *177* | | *153* | *39* | |

Percentages represent weighted results.

## Key stakeholder interview results in full

The following table details all illustrative quotes from the key stakeholder interviews pertaining to the identified themes and sub-themes.

| **Theme** | **Sub-theme** | **Illustrative quotes** |
| --- | --- | --- |
| **Awareness** | Knowledge and understanding | “…the awareness was very, very high” (P14 Government)  “… [the sugar tax] attracted huge attention, which was great.” (P14 Government)  “Doing something as drastic as the sugar tax was, it did generate a lot of conversation in Bermuda.” (P03 Health)  “The ultimate outcome is derived, in theory, to push people towards making healthier food choices. With the overall aim of producing healthier Bermudian population and reducing overall chronic illness. Which is obviously, an individual cost, but also a huge government cost.” (P01 Food and Beverage)  “The whole idea behind the sugar tax was not necessarily to raise a lot of money. But obviously raising money was a by-product of it. But the idea behind it was to raise awareness.” (P02 Health)  “… we were given a push by the Department of Health that [the aim of the sugar tax] would be to combat chronic disease and obesity. It was widely touted as we have a problem with obesity on the island. We have a problem with overweight and obesity in kids and diabetes, and this is a strategy to combat chronic disease.” (P03 Health)  “[The intended aim of the tax was] to encourage people to make healthier choices.” (P04 Government)  “The aim of the tax is to create further disincentives for people to choose sugary options. And conversely, obviously, incentivize consumption of healthier options. The intent of the tax is to motivate different behaviours.” (P09 Government)  “Well, the government ploy was that this was to improve the health of Bermudians. By putting a sugar tax which would presumably make them less likely to buy high-calorie sugary foods.” (P10 health)  “[The aim of the tax was] to raise money for government… Even though it was stated that it would be to create better health outcomes.” (P11 Government)  “I think the aim was to force people to make healthier choices.” (P12 Food and Beverage)  “[The aim of the tax was to] generate more tax revenue for the government…“The aim was to make more money and dress it up as something it is not.” (P13 Food and Beverage)  “One [aim} was to create a dialogue around healthy eating… The second [aim} was to actually raise some money that we were going to use for various health initiatives.” (P14 Government)  “I don't think it's clear, completely, to me. And I certainly don't think it's clear to the public. It's muddied.” (P02 Health)  “There are exemptions from [the sugar tax] which don't seem to make too much sense to me.” (P03 Health)  “…we're not always exactly sure what is going to fall underneath the sugar tax or not. Because sometimes those things do change a little bit.” (P05 Food and Beverage)  “…there was so much confusion.” (P06 Food and Beverage)  “I can't list the items [included in the tax] because I would want to have the tax code in front of me to tell you this.” (P09 Government)  “Not a clue. Not a clue [what falls under the sugar tax].” (P10 Health)  “I mean it was obviously very unclear [what products were subject to the tax] with the law that was passed.” (P12 Food and Beverage) |
| **Beliefs about appropriateness and acceptability** | Taxation as a strategy for health promotion | “As a theoretical construct, I would say it is acceptable. Because there is a recognition that our population is disproportionately unhealthy. And I think within the industry there was support, is some support, for a strategy to deal with that. Because the health-related costs are unsustainable as we look at the next 20 years.” (P01 Food and Beverage)  “We can't sustain our healthcare. Our healthcare budget for diabetes alone last year was in excess of $70 million… If you can think that you could even half that, wow, that would be incredibly important.” (P02 Health)  “People were angry, and they were angry that they felt that they had a right to make a choice. And the government was wrong in trying to put a tax on something where they could make a choice as to whether they wanted to have it or not.” (P02 Health)  “I don't have a problem with the principle of a sugar tax. The design that’s been done in the UK [taxing based on the amount of sugar in SSBs], really good.” (P03 Health)  “I think in some cases [sugar taxes] are a useful design and they can have a positive impact on our health as a population strategy. Yes, so I am in favour of them, [when rolled] out the right way, and in a way that's meaningful, and a way that gives back into programs that will further improve public health.” (P03 Health)  “I am all for a sugar tax of a design on a product, like a sugar sweetened beverage, which you can link to reduced consumption. Which you can then perhaps correlate to change in population health, levels of diabetes or obesity at a later point.” (P03 Health)  “Well [the use of taxes to influence behaviour change] don't work for one thing. They're not effective in changing behavior.” (P06 Food and Beverage)  “I don't think the way to change people's behavior is by trying to beat them over the head with it with taxes. If you want Bermudians to live healthier lives and eat healthy, you have to convince them it's in their best interest… they have to decide on their own. And taxation isn't the way to do that.” (P06 Food and Beverage)  “I think it has a place.” (P08 Health)  “… we definitely think it is the right approach.” (P09 Government)  “… there was a lot of resistance from industry… and to a large extent, from the public.” (P09 Government)  “Waste of time. Complete waste of time.” (P10 Health)  “I'm 100% in favour of health regimes. I'm 100% in favour of control of obesity… By the same token, I also respect people's ability to choose.” (P11 Government)  “If that was the intent [to address health issues], and if the money was ringfenced, then I think that it's quite possible, it's a good and laudable goal, and it's something that I could support. If that was the intent.” (P11 Government)  “I think it needs to be very clear where that money is being put afterwards and to what it has been put to use for. Then I think it's very acceptable. But this is not the case.” (P13 Food and Beverage)  “If you’re not going to teach people why refined white sugar is bad for you or why high fructose corn syrup is bad for you, then why are you, why are you taxing it? There's so much [education] put into alcohol awareness, the negative effects of tobacco. And that's happened over the last 10-20 years. We've seen governments have put in so much money into educating people about the negative side effects. And then whopping them with a big tax. I think that's fine. I think that's perfectly acceptable. Now you make the choice. It's your choice. You know. It's your choice. Nobody's really doing that for sugar.” (P13 Food and Beverage)  “…if you don't actually educate people about it, how can you penalise them for something that most people are completely ignorant on?” (P13 Food and Beverage)  “Well the dialogue [surrounding the sugar tax] was very helpful, I think. It would have been helpful to have that money channeled to…work for chronic disease risk factors. So I'm all for it.” (P14 Government) |
|  | Tax mechanism and level | “There’s no point of sales effect to either that you can say, oh, you know what, that’s cheaper. So I know I should have the diet or the sugar free one, so let me just go buy that because it’s cheaper.” (P03 Health)  “So the wholesalers import the goods and pay the 75%. And then they sell it to the grocery stores. And then the grocery stores sell it to [the consumer]. So then you have got all the different mark-ups or markdowns.” (P04 Government)  “It’s not specifically identified. You know the sugar tax is in there… In Canada, there’s a provincial tax, all these things are tacked on at the end and you see the difference between what was marked on the bottle and what you end up paying. You get to see what the taxes are. Here, it’s just the price on there. You don’t know how much [of the retail price] is the sugar tax.” (P06 Food and Beverage)  “…in most every other jurisdiction where they put in place a quote unquote sugar tax, it is a tax at the point of purchase. So you’re there at the register and you have the option if you’re going to buy a bottle of soft drink, the guy says to you ‘Well, you know, that’s 50% tax on top of the whatever it is you’re going to pay.’ ... and consumers don’t take long to figure this out, they go, ‘Well geez, do I do this, do I not?’ In Bermuda, it’s imposed at the port of entry… We raise our price, we pass it on to retailers, they mark it up, pass it on, and the consumer pays more… It gets all murky.” (P7 Food and Beverage)  “We decided quickly that we weren't going to be able to [administer the tax] at point-of-sale because we don't have any tax structure that would allow that. And it would also, if we did put in infrastructure to facilitate that, it would wipe out any benefit of that the tax. It would be very expensive to implement.” (P14 Government)  “I mean [Bermuda] kind of did the sugar tax using the old tax, you know just using the tariff, the import tariff. And I think that’s one of the reasons why it’s maybe fallen short. It is because nobody’s done the heavy lifting that’s really required.” (P14 Government)  “I was surprised to see rolled out in the way it was, to the extent of the amount of the tax and the fact that they've done it across the board [taxing food items as well as sugar-sweetened beverages].” (P03 Health)  “I think we're the only country in the world that has a rate this high.” (P04 Government)  “The import duty on a sugary drink, I think is more than cigarettes, more than tobacco.” (P06 Food and Beverage) |
|  | Intervention targets | “As the process evolved, and we rolled into phase two, which was then 75%, it became more ambiguous. A number of items that did not fall within the duty rates were being included, it became much more onerous and a little bit more frustrating… because we were having items dutied that in our opinion had nothing to do with what the sugar tax should have been focusing on. Things like protein powders and smoothies were being hit with the tax.” (P01 Food and beverage)  “… [The inclusion of food products] diluted the message…” (P02 Health)  “When I've reviewed studies… the majority of countries were putting a sugar sweetened beverage tax on, where it doesn't get complicated with starting to tax foods.” (P03 Health)  “So [the government] were sort of dipping into a cross-section [of items]. We won’t touch cereals, but we will do granola bars. So you know it went across the board…” (P03 Health)  “…once [it was] realized that white chocolate was being taxed, simply because it fell in that category with the candy… [all chocolate had to be added] because it just doesn't make any sense. So that's why we ended up adding the other chocolate.” (P04 Government)  “…imported cookies were not taxed. Even though they have just as much, or more, sugar than the locally baked ones. So they disadvantage local bakers… They didn't think it through and didn't ask for any help.” (P06 Food and Beverage)  “Initially, they hadn't even considered diet sodas.” (P06 Food and Beverage)  “And what was so confusing or frustrating, was that it didn't matter if it was 50 grams of sugar per serving, or 0.5 grams of sugar per serving.” (P07 Food and Beverage)  “There was also a major flaw at the very outset that because the way the tax code was written, the diet sodas ended up taxed as well.” (P09 Government)  “So, the question begged as to how carefully the implementation and the determination as to what categories would be covered. I don't believe that was well thought through.” (P11 Government)  “…most countries that bring in a sugar tax tend to put some sort of tier level in place for how much sugar is contained in an item.” (P13 Food and Beverage) |
| **Beliefs about economic and equity impacts** | Impact on prices | “We have, I would say absorbed some impact of it. Because realistically speaking… passing along the full magnitude of the increase in some areas would make the product unsellable.” (P01 Food and Beverage)  “I didn't see a difference in the sweet drinks. Particularly, I was looking at like iced teas and unsweetened iced teas and they were all, a beverage would be you know $3.60 - $4 depending on what it was. The sugar free or the sugar free version. It wasn't cheaper.” (P03 Health)  “But that food right beside the one that was taxed when it landed, has gone up to absorb the price of the one that was taxed at 75%. So they have all gone up.” (P03 Health)  “… [the] cost of a grocery basket of items, it went up significantly. All of it. Whether it was an item that was listed, by ingredient, to be one that would be penalized by the sugar tax, or not. It’s raised the cost of everything.” (P03 Health)  “…to me, everything has gone up.” (P04 Government)  “You can’t pass {the full amount of sugar tax] on. It’s not fair to the customers…” (P05 Food and Beverage)“And you know, honestly, it wasn't really a 75% increase. If you look at the true numbers, sugary [beverages] were already being taxed at 25% anyways. They moved it up to 75% so there was a [change of] 50% that actually jumped up.” (P05 Food and Beverage)  “…the sugar tax, it just made the price of all foods go up. That’s my personal experience and I’ve heard other people say the same thing.” (P08 Health)  “… the supermarkets raised the prices on almost everything.” (P08 Health)  “[Retailers] just raised all prices. They didn’t do it in a way that reflected the tax.” (P09 Government)  “…the one massive weakness that we saw is that retailers – wholesalers and retailers – in their lack of support for the sugar tax, are not differentiating prices on the shelf sufficiently. By the time the consumer goes to the shelves to buy whatever product, the way that the cost was spread by the wholesalers and the retailers, creates virtually no price differentiation. In some instances, there is zero price differentiation between a diet soda and a regular soda.” (P09 Government)  “And it did find about a 15% difference [in the price of sugar taxed item compared to a non-sugar-taxed item]. I’m speaking orders of magnitude, right. It may not be exactly 15%, but it was around, overall, altogether around the 15% difference.” (P09 Government)  “I believe, just judging from the prices that I’ve seen across the board and how my own grocery cart has been impacted, I just think that it’s been an across the board this is the cost of imports of this container, or this kind of good, and therefore let’s mark it up.” (P11 Government)  “What it has done though, having the sugar tax, is to just create almost running rampant the cost of groceries, period. Sugar or not sugar, this seems to be the excuse to be able to increase costs, across the board.” (P11 Government)  “Obviously, things have become incredibly more expensive, certain items.” (P13 Food and Beverage)  “…if you’d seen a real impact… it would have gotten a lot more expensive [to buy soda and sweet things], and I don’t believe that it has.” (P14 Government)  “…it did look like some merchants were trying to spread the tax burden across a whole bunch of items in their stores.” (P14 Government) |
|  | Affordability of healthy food | “If a bag of apples is still going to be $9, then a Snickers bar at $1.85 is still going to be less expensive and may go into a kid’s lunchbox instead.” (P01 Food and Beverage)  “I generally just find goods in Bermuda to be expensive anyway, especially in the grocery store.” (P04 Government)  “…a lot of our juices, they may have a fraction of sugar in them. They are still considered diet, they say diet on them, but those are still sugar taxed.” (P05 Food and Beverage)  “Everything on this island, it’s expensive. Not just the healthy food, the junk food is expensive.” (P08 Health)  “I think it’s ridiculous that you’re looking at a bag of clementines that only contains eight or nine tiny little oranges. And so divide that into almost $12.” (P08 Health)  “Well, healthy food is expensive.” (P10 Health)  “Because obviously a healthy soda water that has a little bit of fruit juice added, now has traces of sugar in it that falls under the sugar tax and therefore becomes more expensive overall.” (P12 Food and Beverage)  “I think that’s one of the biggest failures of the sugar tax, in general, was that it wasn’t promoting switching to lower sugar variants… [Drinks that] have less than 1% sugar in them, but they’re still hit with the sugar tax. So at the end of the day if you really look at trying to push somebody from a [high sugar soda] to a one gram [of sugar sparkling water], it’s very difficult to do that when they’re both hit with the same tax.” (P13 Food and Beverage) |
|  | Impact on socioeconomic equality | “[The sugar tax] is, in many ways, disproportionally targeting the population that you’re trying to have benefit from it from a socioeconomic standpoint. Because health related conditions, as well as outcomes, are obviously tied to socioeconomic standing. So the people who are eating an unhealthy diet, and maybe making less wise choices, are not necessarily going to change that behavior. But it’s going to cost them more out-of-pocket resources, which is going to put them in even a worse position.” (P01 Food and Beverage)  “I guess I am always confused about the argument that it’s the poor people that are being affected the most. I find that a little bit, almost insulting, if I was in the lower economic sector… I don’t understand why people feel that it’s been the poor people who’ve been affected the most.” (P02 Health)  “So I think that this has become my concern with it… [that the sugar tax has] become rather more of a regressive tax than a progressive tax.” (P03 Health)  “People on limited budgets definitely should and are feeling the pinch with the raise of the prices because of the sugar tax.” (P03 Health)  “…all you’re going to do by increasing the duty on [sugar-taxed items], is you’re going to increase the ultimate price. And that’s going to disadvantage the, for lack of a better term, the poorer consumer. Because people who only have a certain amount of disposable income, spend a disproportionately large amount on food and beverage.” (P07 Food and Beverage)  “[Those] most affected [by the sugar tax] are the people who can ill afford it. Because people having determined what’s appropriate to them for the lifestyle that they want, and the comforts that they seek, are finding themselves having to pay more.” (P11 Government)  “But it’s the people who are perhaps on financial assistance, people who are just about struggling to make ends meet, people who have children to educate, people who have, mortgages to pay and the like. And then the groceries on top of it. So when you put all of that into the equation and some way, somehow, you find that the people who are most impacted [by the sugar tax] are the people who are effectively on the lower end of the earnings spectrum.” (P11 Government)  “…from a financial position you’re making people worse off.” (P01 Food and Beverage)  “But [a sugar tax] doesn’t change behavior, just increases the cost of living.” (P06 Food and Beverage)  ” …it makes everything more expensive.” (P06 Food and Beverage)  “It raises the cost of living in Bermuda.” (P07 Food and Beverage) |
|  | Use of tax revenues | “So my suspicion is it’s basically a million and a half dollars or whatever [the government raised from the sugar tax] thus far just going into the general government coffers. And likely to remain that way in the short term, at least, while they’re dealing with the existing government deficit issues.” (P01 Food and Beverage)  “Yeah, from the proposed perspective, a magic unnamed amount was supposed to be targeted towards public education and consumer behavior. We haven’t seen any evidence of that.” (P01 Food and Beverage)  “Well, we were told that [revenue from the sugar tax] would be used for health-focused programs. There was no clear indication as to who would be doing those programs… But my understanding is that [the revenue] has not filtered down to anybody.” (P02 Health)  “Just [used] I think to swallow up the debt.” (P03 Health)  “The $1 million, $3 million that [the government] grossed in the first year [did not come] back directly into any specific new [health education] curriculum.” (P03 Health)  “The government’s official position is still that a portion of [the sugar tax revenue] will be allocated to certain health programs. My personal belief is that it’s going into the big pot and it’s never going to happen.” (P06 Food and Beverage)  “…originally government said a portion of the proceeds from the sugar tax would go to health initiatives. When they got nailed about that then they came back and revised the wording. But there are any one of a number of health organizations which are still waiting to get any kind of extra stipend from the government… The reality is that the money seems to be disappearing into the general fund and not going to help health initiatives.” (P07 Food and Beverage)  “With the sugar tax, it’s not [ring-fenced money]. It’s just tax revenue that goes into the tax coffers.” P09 Government  “[Sugar tax revenue] goes into the same fund that all of the government tax and revenue money goes in.” P09 Government  “Well what the government, and the Premier specifically, committed to was that all of [sugar tax revenue] would be used for health initiatives. Or health promoting initiatives. Or initiatives that would advance health… That side of things has not panned out quite as intended.” (P09 Government)  “…and as we knew would happen from the beginning… [all the sugar tax] money has gone into the Consolidated Fund.” (P11 Government)  “I don’t think anybody actually knows where that money is going. It’s going into the coffers somewhere.” P12 (Food and Beverage)  “…it was decided that there were other more worthy causes to put [sugar tax revenue] towards [other than health initiatives].” (P14 Government)  “…the money didn’t actually go to health initiatives...” (P14 Government) |
| **Beliefs about effectiveness** | Impact on purchasing and consumption of sugar-taxed products | “…there are particular areas where we’re selling sort of significantly less. But they’re really more on the on the fringe, they’re not the core items. We’re not selling less chocolate, necessarily, because of it. We’re not selling certainly less refined bags of white sugar or icing sugar because of it. We are not selling less candy because of it… Our number of individual chocolate bars that we sell isn’t being impacted.” (P01 Food and Beverage)  Do I think it’s going to work in terms of changing consumer behavior? It might at the edges. And it will only work if there is a concerted consumer campaign around it.” (P01 Food and Beverage)  “If I want a Coke, I’m going to have a Coke.” (P01 Food and Beverage)  “But it’s naive to say that the implementation of the sugar tax has had a massive benefit because people buy less sugary sodas. That’s been on the decline for 10 years. It may have helped accelerate it, but it’s simplistic to attribute all of the decline in the last 18 months to something that’s already been transformational for 10 years.” (P01 Food and Beverage)  “…we’re always we’re always looking for healthier alternatives as a general rule or trying to find better alternatives. That’s complicated by the fact that ultimately the consumer will have a large role in what we can bring in and be successful bringing in.” (P01 Food and Beverage)  “I don’t know if it’s going to have that much of an effect on people buying stuff unless the prices are that much higher.” (P04 Government)  “… if you're in a store, and you're looking at regular soda, and I'm just saying diet soda, and they're both the same price, there's no incentive to buy the diet.” (P04 Government)  “[Purchasing of sugar-taxed items] stayed pretty consistent. Like, people want what they want.” (P05 Food and Beverage)  “You know, we have definitely increased a lot more sugar-free or non-sugary items…to try to find ways to still present a great quality product to customers, without having to pay the 75% duty on those particular items.” (P05 Food and Beverage)  “We sell what people buy. People buy what they want, not what the government taxes them on.” (P06 Food and Beverage)  “There has been some minor shifting [of consumption towards non-sugar beverages]. Minor. Other than that, the trends remained the same. [Other] products that are full sugar, 75% duty, that are growing in double digits. Because consumers want them. They don’t care what the price is. Tax makes no difference to their consumption at all.” (P06 Food and Beverage)  “And we did observe through surveys that consumption of sugary items of sodas in particular for which we had benchmark data had gone down.” (P09 Government)  … whenever you do an initiative of this nature it generates noise in the community, it makes people talk about the issues that you’re trying to tackle, hopefully… The education that it generates in the public, it generates conversation, right? And that conversation can lead to changes in behaviour, quite importantly.” (P09 Government)  “I don’t think it’s going to make a… bit of difference.” (P10 Health)  “…I don’t know how much water has replaced sweet drinks. I don’t know what the impact of that replacement, if significant, has been on what [was] indicated to be the desired outcome. So I really honestly don’t have a clue...” (P11 Government)  “… nobody is going to tell me that if you have to pay more for your soda, and you pay more for your candies, that you’re going to forego those choices and buy some carrots…If you want soda, then you want soda. If you want carrots, you’re going to buy carrots.” (P11 Government)  “We’ve never researched it, but we don’t believe people have made different choices because of [the sugar tax].” (P12 Food and Beverage)  Yeah, I don’t see anybody switching their habits because of the sugar tax. Obviously, things have become incredibly more expensive, certain items. We don’t see customers shifting to non-sugar items.” (P13 Food and Beverage)  “People have just gotten used to paying whatever they’re paying for their sweet drink. Yeah, I’m not sure it has actually caused any lasting behaviour change.” (P14 Government)  “It changed some behaviour for a short period of time.” (P14 Government) |
|  | Impact on health-related outcomes | “I guess if [the sugar tax] legitimately impacted consumer behavior, then potentially, there would be benefits towards reduced diabetes and better long-term health outcomes. That’s a separate question to whether or not I think it does that.” (P01 Food and Beverage)  “…I don’t think there’s been a comprehensive follow up to how many people have stopped drinking sodas compared to those who continue to drink them.“ (P02 Health)  “If we had specifically designed programs and further education that were as a result of the revenue from [the sugar tax], then perhaps we’ll see an effect of that in improving health.” (P03 Health)  In Bermuda, for example, they don’t have healthy school dinners. [Is the government] going to start implementing a healthy school meal program? But what are they going to do? We didn’t have any idea of how [the sugar tax] was going to improve our health with the money raised. (P03 Health)  “I can’t speak to any health impacts. It would be good to know whether there have been any.” (P11 Government)  I think that the [health] outcome could be a lot better if there had been a thought process behind what was done and not just a monetary intent behind what has been done in respect to the sugar tax.” (P11 Government)  “It was not well thought through, not well implemented; and therefore, it’s our opinion that it has none of the advertised effect on the public health that it was supposed to.” (P12 Food and Beverage)  “I don’t think it will [have an impact on health] …it’s not a sustainable change for changing people’s behaviour around chronic disease risk factors.” (P14 Government) |
|  | Impact of the healthy food subsidy | “But I am challenged when I listened to the government talking about reducing the duties on certain products. Because when [the government] say they have reduced the duties on cauliflower or broccoli or something like that, it literally amounts to three or four cents a pound. Half of the products that they talk about are embargoed six to eight months a year. So there is no impact at all.” (P01 Food and Beverage)  “The percentage of the duty as it relates to the eventual sell price is pretty minimal. So if you’re talking two cents an apple, really, by the time it gets to a retail level. No one is going to make a decision based off of that.” (P01 Food and Beverage)  “Interesting enough, I think carrots were on the list [of items receiving a reduced import duty]. And carrots are not something that’s imported anyway… we’ve never imported carrots. There’s an embargo on those since I was [very young]. So why are we putting them on the list? …I don’t think it transcribes into anything meaningful in the grocery store [by way of a lowered price].” (P03 Health)  “So in the first phase, this was sort of like a give and take thing from the government. They lowered the duty rate to 0% on healthy items such as chicken eggs, potatoes, cauliflower, broccoli, turnips, oranges and apples. But I will say that the duty rate on them before lowering it to zero was only 5%... Now, did that change the price of the items in the stores? No.” (P04 Government)  “[Fruits and vegetables are] all going to be transported thousands of miles by land, and thousands of miles by ocean, and all that uses fuel, [the cost of] which goes up and down. There were too many factors for the 5% duty drop to make a lot of difference...” (P06 Food and Beverage)  “One thing I don’t know if a lot of consumers are aware of, is that when Bermuda grown vegetables and fruit, especially vegetables, are in season, there is an embargo placed on import. So when Bermuda has got plenty of broccoli, fresh broccoli, when the guys are harvesting, they stop import.” (P07 Food and Beverage)  “Because the duty rate is only 5%, wiping that out has a very negligible impact on the final retail price… There are seasonal pressures and crop pressures on prices that have nothing at all to do with the import duty.” (P07 Food and Beverage) |
